# Rupture of the Aorta

**Published:** 1885-06

**Authors:** Augustin Prichard


### RUPTURE OF THE AORTA. By Augustin Prichard, F.R.C.S.

I was sent for in the afternoon of April 4th to see Mr. A. B. C., one of my ordinary patients, who was described in the message as being very ill. He was 58 years of age, moderately stout, but pallid in complexion, a very temperate man, leading a regular life. He generally walked down into Bristol from the farther side of Clifton, and back again, but took very little other walking exercise beyond his garden; and he drove about the country not unfrequently. He had been subject to an occasional fainting fit, and to severe attacks of colic; but for a considerable time he had been free from either of these ailments. He was one of a large and healthy family, and his father and mother are still living.

On the 2nd of April he went down to Bristol as usual, and walked back again, and was apparently in his ordinary health. On the 3rd, he dined on fish, eating a small hard piece of roast beef afterwards, rather to keep company with others at his table than from any desire for more food; and he spent the evening as usual, and went to bed in very good spirits, and apparently well.

In the middle of the night, he was turning round in bed, when he felt something give way in his chest, and said he had strained himself. He became sick and faint, and continued so until I saw him, about four o'clock in the afternoon. He was then vomiting a thin bloody fluid, and had been able to keep down no nourishment; he was cold, and rather collapsed and restless, throwing his arms about like a patient suffering from the effect of hemorrhage. Under the idea that he was suffering from one of his old attacks of colic, I prescribed for him a calomel pill, and some bismuth. He was not sick again after taking the pill, and he said he felt much better, and took some light nourishment. He took his bismuth mixture once in four hours, until one o'clock in the morning of the 5th, when he said he was better, and got out of bed easily. When he was visited, at eight in the morning, he was found dead, and beginning to get cold; but the dose of his medicine due at five a.m. had been taken, and he had put out the candle (at that time in the dawn of the morning), and returned to bed, and had apparently died quietly in his sleep.

*P. M. E.*—There was a large quantity of fat about the body. The lungs were healthy; the pericardium was found to be distended with an enormous coagulum, surrounding the heart on all sides; and, when it was turned out, a rent in the convexity of the arch of the aorta, just at its commencement, was discovered. The rupture was an inch and a quarter long, and very ragged through the external and middle coats of the vessel; but the division of the inner lining coat was a clean, straight line, as if it had been cut. There was no special dilatation of the artery, but it was remarkable for the thinness and softness of its tissue, and some very small white spots, apparently fat, were deposited on the inner surface.

There was no sign of a general atheromatous state. The heart was very large and flabby, and much loaded with fat externally. The valves were healthy, but the right ventricle was dilated, and its walls much thinned, so that, on a section, the divided muscular fibre formed a very thin layer, compared with the stratum of fat on the outside; and some parts of the fleshy tissue were of a white colour from fatty degeneration. The other organs, as far as they were examined, were sound.

I believe that the first rent in the outer coats of the aorta occurred when the patient was turning round in bed (and he had tenderness, on pressure over the part, when I saw him), and the sickness followed. The blood which escaped from the stomach could have had no direct source in the ruptured vessel, but was due to the straining efforts to vomit, and these efforts were induced by the laceration. It is probable that a little leakage was going on into the pericardium all the time from the first attack of pain, and that soon after he got back into his bed, at five o'clock in the morning, the inner membrane of the aorta gave way by a sudden split, and he died immediately.

